# Chemical genetic screens reveal defective lysosomal trafficking as synthetic lethal with NF1 loss

**DOI:** 10.1242/jcs.262343

**Published:** 2024-08-14

**Authors:** Stephanie J. Bouley, Andrew V. Grassetti, Robert J. Allaway, Matthew D. Wood, Helen W. Hou, India R. Burdon Dasbach, William Seibel, Jimmy Wu, Scott A. Gerber, Konstantin H. Dragnev, James A. Walker, Yolanda Sanchez

**Affiliations:** ^1^ Department of Molecular and Systems Biology, Geisel School of Medicine, Hanover, NH 03755, USA; ^2^ Center for Genomic Medicine, Massachusetts General Hospital, Boston, MA 02114, USA; ^3^ Department of Biochemistry and Cellular Biology, Geisel School of Medicine, Hanover, NH 03755, USA; ^4^ Department of Pharmacology and Toxicology, Geisel School of Medicine, Hanover, NH 03755, USA; ^5^ Cincinnati Children's Hospital, University of Cincinnati, Cincinnati, OH 45229, USA; ^6^ Department of Chemistry, Dartmouth College, Hanover, NH 03755, USA; ^7^ Department of Medicine, Geisel School of Medicine, Hanover, NH 03755, USA; ^8^ Section of Medical Oncology, Geisel School of Medicine, Hanover, NH 03755, USA; ^9^ Dartmouth Cancer Center, Dartmouth-Hitchcock Medical Center, Lebanon, NH 03766, USA; ^10^ Department of Neurology, Massachusetts General Hospital, Harvard Medical School, Boston, MA 02114, USA; ^11^ Cancer Program, Broad Institute of MIT and Harvard, Cambridge, MA 02142, USA

**Keywords:** NF1, RAS, Synthetic lethal, Mitochondria, Lysosomes, BORC

## Abstract

Neurofibromatosis type 1, a genetic disorder caused by pathogenic germline variations in *NF1*, predisposes individuals to the development of tumors, including cutaneous and plexiform neurofibromas (CNs and PNs), optic gliomas, astrocytomas, juvenile myelomonocytic leukemia, high-grade gliomas and malignant peripheral nerve sheath tumors (MPNSTs), which are chemotherapy- and radiation-resistant sarcomas with poor survival. Loss of *NF1* also occurs in sporadic tumors, such as glioblastoma (GBM), melanoma, breast, ovarian and lung cancers. We performed a high-throughput screen for compounds that were synthetic lethal with *NF1* loss, which identified several leads, including the small molecule Y102. Treatment of cells with Y102 perturbed autophagy, mitophagy and lysosome positioning in *NF1*-deficient cells. A dual proteomics approach identified BLOC-one-related complex (BORC), which is required for lysosome positioning and trafficking, as a potential target of Y102. Knockdown of a BORC subunit using siRNA recapitulated the phenotypes observed with Y102 treatment. Our findings demonstrate that BORC might be a promising therapeutic target for *NF1*-deficient tumors.

## Teaser

**Highlighted Article:** We screened for compounds that were synthetic lethal with NF1 loss and identified BORC, a complex required for lysosome trafficking, as a promising therapeutic target for *NF1*-deficient tumors.

## INTRODUCTION

RASopathies are a group of rare genetic conditions defined by germline alterations in the RAS-MAPK pathway that often result in constitutive activation of the phosphoinositide 3-kinase (PI3K), RAF and mammalian target of rapamycin (mTOR) pathways located downstream of RAS (Ratner and Miller, 2015). Together, these pathways drive translation, cell proliferation, cell motility and cell invasion. One of the most common RASopathies with an incidence rate of ∼1:3500 is neurofibromatosis type I (NF1) (Walker and Upadhyaya, 2018). NF1 is an autosomal dominant tumor-predisposing syndrome categorized by the loss of neurofibromin (NF1) protein expression due to genetic mutation or other alterations in the *NF1* gene. NF1 is a GTPase-activating protein (GAP) that negatively regulates the activity of RAS by stimulating its intrinsic GTPase activity. Loss of NF1 thereby results in RAS hyperactivation and aberrant activation of downstream pathways (Simanshu et al., 2017). Elevated RAS signaling leads to the formation of non-malignant tumors including cutaneous neurofibromas (cNFs) and plexiform neurofibromas (PNs) in NF1. cNFs are considered the largest tumor-related burden in NF1 individuals, with no effective therapy options (Allaway et al., 2018). PNs occur along peripheral nerves and can predispose individuals with NF1 to the development of malignant peripheral nerve sheath tumors (MPNSTs) and at an earlier age than the general population (Avery et al., 2017; Korf, 2000; Yao et al., 2023). MPNSTs are highly aggressive radiation- and chemotherapy-resistant tumors with a 5-year survival rate of 15–50% (Farid et al., 2014). Individuals with NF1 can also develop neoplasias including optic nerve gliomas, pilocytic astrocytomas, juvenile myelomonocytic leukemia, gastrointestinal stromal tumors and both low- and high-grade gliomas (Ly and Blakeley, 2019). Loss of *NF1* also occurs in 5% of all sporadic tumors, including glioblastoma multiforme (GBM), breast cancer, endometrial cancer, ovarian cancer, both melanoma and non-melanoma skin cancers, and lung cancer (Philpott et al., 2017). Using genetically engineered mouse models, *NF1* loss has been shown to be a driver of GBM (Alcantara Llaguno et al., 2015; McGillicuddy et al., 2009). In tumors with sporadic *NF1* loss, progression-free survival is significantly lower in *NF1*-deficient individuals compared to other groups for non-small cell lung cancer and acute myeloid leukemia, making *NF1* loss a poor prognostic factor (Giraud et al., 2023). Based on this information, there is a significant need to identify novel therapeutic targets in *NF1*-deficient tumors associated with the condition NF1 as well as tumors with sporadic *NF1* loss.

Over the past two decades, significant steps have been taken to address the lack of therapeutic options for those with *NF1*-deficient tumors, with a focus on targeting RAS effector proteins (See et al., 2012). Clinical trials have focused on repurposing cancer drugs have been carried out in patients with PNs using RAF, mTOR and MEK inhibitors (Walker and Upadhyaya, 2018). Although these inhibitors have had varying degrees of success, there are limitations associated with each. RAF inhibitors such as dabrafinib have been shown to be effective in *BRAF*-mutant melanomas; however, in *BRAF*-mutant melanomas with concurrent *NF1* mutations, RAF inhibitors are less effective due to remaining hyperactive RAS signaling (Maertens et al., 2013). mTOR inhibitors are also effective in tumor types associated with RAS dysregulation; however, feedback signaling mediated by S6 kinase can increase AKT signaling, bypassing the inhibitory effects of the mTOR inhibitor (Sabatini, 2006). The combination of mTOR and PI3K inhibition is able to prevent this negative feedback loop but remains ineffective at inhibiting the RAF-MEK-ERK pathway (See et al., 2012). Work with MEK1/2 inhibitors such as selumetinib is encouraging and effective in the majority of NF1 participants with PNs or low-grade gliomas, and the drug was recently granted FDA approval for the treatment of NF1 PN (Dombi et al., 2016; Gross et al., 2018, 2020). Although this discovery has made a significant impact on the clinical maintenance of NF1, there are several compounding factors associated with MEK inhibition. First, selumetinib has only been successful in NF1 pediatric patients with PNs; treatment does not alleviate other tumor burdens, such as cNFs, nor is selumetinib approved at this time for adult NF1 patients (Solares et al., 2021). Additionally, NF1 patients with long-term exposure to MEK inhibition develop side effects including peripheral edema, hair color change, nausea, headaches, a variety of skin conditions, blurred vision and cardiac abnormalities (Baldo et al., 2021; Gross et al., 2023; Klesse et al., 2020). Therefore, there still exists a need for additional therapeutic targets for *NF1*-deficient tumors.

To identify novel pathways to target *NF1*-deficient tumors, we developed an unbiased high-throughput synthetic lethal screening approach utilizing a library of tool compounds and a budding yeast model system of *NF1* deficiency. We discovered several promising compounds through this screen and previously reported the target or the mechanism of action of two of these (Allaway et al., 2017; Wood et al., 2011). Here, we describe the mechanism of action of a third compound, Y102, and identify inhibition of lysosomal trafficking as a potential vulnerability of both sporadic and neurofibromatosis type I-associated *NF1*-deficient tumors.

## RESULTS

### Identification of tool compound Y102 as synthetic lethal *in vitro* with loss of NF1 protein

We developed and conducted a high-throughput screen in *Saccharomyces cerevisiae* lacking a yeast homolog of NF1, IRA2 (*ira2Δ*) to identify tool compounds that elicited synthetic lethality with *NF1* loss (Wood et al., 2011). *ira2Δ* yeast have increased RAS-GTP, which results in increased MAPK and protein kinase A (PKA) signaling, which is analogous to the pathways that are activated in *NF1*-deficient Schwann cells (Kim et al., 2001). The yeast strains used in these screens also lacked the *ERG6* gene to facilitate drug retention (Emter et al., 2002).

**Fig. 1.**
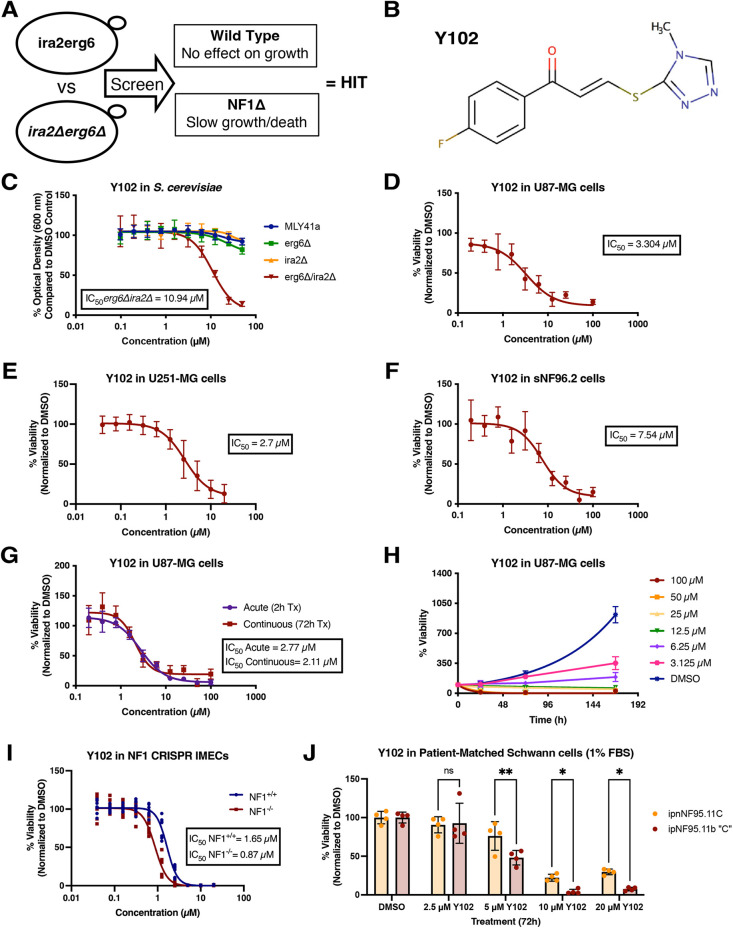
**Y102 is synthetic lethal in *NF1*-deficient yeast and exhibits activity in *NF1*-deficient human cell lines.** (A) Schematic of screen design. The efficacy of compounds was compared between *erg6Δ* and *erg6Δira2Δ* yeast. Compounds were considered hits if *erg6Δira2Δ* yeast exhibited slow growth or death at concentrations that had no effect on the growth of *erg6Δ* yeast. (B) Chemical structure of small molecule Y102. (C) Yeast were grown at 30°C starting at an OD_600_ of 0.05 with Y102 at concentrations ranging from 100 µM to 0.039 µM. At 18 h, OD_600_ was measured. (D) U87-MG cells were treated for 72 h with Y102 at concentrations ranging from 100 µM to 0.039 µM. (E) U251-MG cells were treated for 72 h with Y102 at concentrations ranging from 20 µM to 0.020 µM. At 3 h prior to collection, cells were stained with alamarBlue. (F) sNF96.2 cells were treated for 72 h with Y102 at concentrations ranging from 100 µM to 0.039 µM. (G,H) U87-MG cells were treated continuously for 72 h, or acutely for 2 h followed by washout, for up to 3 days with Y102 at concentrations ranging from 100 µM to 0.039 µM. (H) Results are normalized to day zero plating. (I) Wild-type (*NF1^+/+^*) and mutant (*NF1^−/−^*) IMECs were treated for 72 h with Y102 at concentrations ranging from 20 µM to 0.020 µM. (J) ipnNF95.11C (*NF1^−/+^*) and ipNF95.11b ‘C’ (*NF1^−/−^*) cells were treated in 1% FBS-containing growth medium for 72 h with 20 µM, 10 µM, 5 µM, or 2.5 µM Y102. ns, not significant (*P*>0.05); *P<0.05; **P<0.005 (two-way ANOVA with multiple comparisons using the Tukey correction method). Viability was measured using Hoechst 33258 unless otherwise stated. For C–G,I,J, results are normalized to DMSO. Results in C–H are the mean±s.d of three experiments; I is the mean of two experiments (four technical replicates each). J is the mean±s.d of four technical replicates.

We hypothesized that a synthetic lethal screen with yeast cells that lack an *NF1* ortholog would identify tool compounds that might be selective for *NF1*-deficient human tumor cells. We screened >5100 structurally diverse compounds selected from and representative of a >340,000 drug-like compound library housed by the University of Cincinnati Drug Discovery Center (Wood et al., 2011). In this screen, a compound was considered a hit if *ira2Δ* yeast exhibited slow growth or death at concentrations that had no effect on the growth of the control strain (Fig. 1A). Utilizing this approach, Y102, a vinylogous thioester tool compound, was found to have an inhibitory effect on the *erg6Δira2Δ* yeast at concentrations that had no effect on the control strains (Fig. 1B,C). To determine whether Y102 was also effective in human tumor cells, the efficacy of Y102 was evaluated in two *NF1*-deficient human cell line models of glioblastoma, U87-MG and U251-MG, and an *NF1*-deficient MPNST cell line, sNF96.2. U87-MG is recognized as a human glioblastoma cell line of unknown origin and has reduced levels of NF1 due to elevated proteasomal degradation as opposed to possessing an *NF1* mutation (Allen et al., 2016; McGillicuddy et al., 2009). We found that all three *NF1*-deficient cell lines were sensitive to Y102 (Fig. 1D–F). We also found in U87-MG cells that acute treatment of 2 h resulted in the same degree of growth inhibition compared to continuous treatment for 72 h (Fig. 1G). To understand the long-term efficacy of Y102 treatment *in vitro*, we investigated the effect of Y102 on cell proliferation after 7 days of treatment. Cell proliferation was inhibited by continuous Y102 treatment with increasing concentrations starting at 3.125 µM Y102 (Fig. 1H). The selectivity observed in our yeast model of *NF1* loss was recapitulated in *NF1*-deficient immortalized mammary epithelial cells (IMECs) (DiRenzo et al., 2002). CRISPR/Cas9-mediated gene editing was used to generate indels in exon 2 of *NF1* in IMECs. Single-cell clones were screened for *NF1* loss by western blotting (Fig. S1). *NF1*-deficient IMECs were found to be more sensitive to Y102 than the isogenic parental wild-type line (Fig. 1I). Additionally, we utilized a pair of *NF1*-deficient Schwann cells derived from individuals with NF1 (ipnNF95.11C and ipNF95.11b ‘C’) to demonstrate differences in sensitivity between cells with loss of one *NF1* allele and two alleles (Li et al., 2016). We found that Schwann cells derived from a PN were more sensitive to Y102 compared to the matched heterozygous counterpart at multiple concentrations of Y102 (Fig. 1J).

### Y102 results in increased expression of autophagy and oxidative stress markers

To identify the mechanism of action of Y102-mediated cell death, we examined expression of proteins associated with DNA damage, apoptosis, proteasome inhibition and autophagy (also referred to as macroautophagy) pathways (Lavrik et al., 2005; Liu et al., 2016; Sharma et al., 2012). First, we examined the expression of the DNA damage marker γ-H2AX, a histone H2A variant that is phosphorylated following the generation of double- and single-stranded DNA breaks (Sharma et al., 2012). There was no significant change in γ-H2AX expression following Y102 treatment (Fig. S2A), suggesting that Y102 does not mediate cell death through a DNA damage response pathway. We next examined apoptotic cell death following Y102 treatment by monitoring the cleavage of caspase-3 (Lavrik et al., 2005). We did not observe a difference in the amount of cleaved caspase-3 between Y102 treatment and vehicle control (Fig. S2B). This result suggests that Y102 does not mediate cell death through caspase-3-mediated apoptosis, and was confirmed by western blotting for PARP (also known as PARP1), an enzyme that is cleaved by caspase-3 under apoptotic conditions (Fig. S2C) (Chaitanya et al., 2010).

The ubiquitylation of proteins can mark them for degradation mediated by the proteasome, the unfolded protein response or autophagy, triggering cell death. To determine whether Y102 acts as a direct inhibitor of the proteasomal active sites, we utilized the activity-based broad-range fluorescent inhibitor MV-151, which can bind to and inhibit all three subunits of the proteasome (Verdoes et al., 2006). We performed a competitive binding assay in which cells were treated with DMSO, Y102 or a combination of proteasome inhibitors to serve as a positive control, and the lysates were incubated with MV-151, which can only bind in the absence of proteosome inhibitors. Inhibition of proteasomal subunits was only observed following treatment with the positive controls and not with Y102, as indicated by the presence of MV-151 in the Y102-treated sample, eliminating proteasome inhibition as a mechanism of action of Y102-mediated cell death (Fig. S2D).

**Fig. 2.**
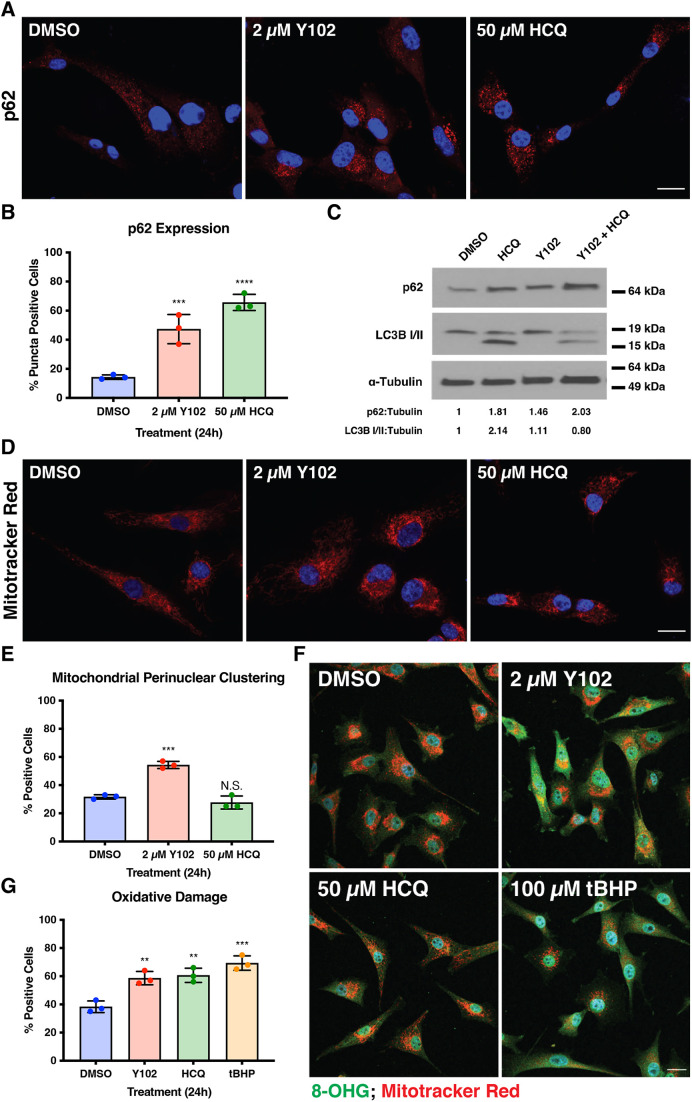
**Y102 treatment results in increased expression of autophagy and oxidative stress markers and alters the mitochondrial network.** (A) U87-MG cells were treated with DMSO, 2 μM Y102 or 50 μM HCQ, an autophagy inhibitor for 24 h. Cells were stained for the autophagy marker p62 (red). DAPI was used to counterstain cell nuclei (blue). Scale bar: 50 µm. (B) 100 cells as in A were analyzed for puncta in triplicate experiments. (C) Western blotting analysis of autophagy markers following a total of 24 h treatment with 2 µM Y102, 50 µM HCQ or the equivalent amount of DMSO (<1%) or 12 h of 2 µM Y102 followed by an additional 12 h with 50 µM HCQ present. Densitometry analysis was used to determine the ratios of p62 and LC3-I and II to α-tubulin control. Values are for blot shown. Blot is representative of three experiments. (D) U87-MG cells were treated with DMSO, 2 μM Y102 or 50 μM HCQ for 24 h. At 30 min prior to the end of treatment, cells were stained with MitoTracker Red CMXROS (red). DAPI was used to counterstain cell nuclei (blue). Scale bar: 50 µm. (E) 100 cells as in D were analyzed in triplicate experiments to determine differences in perinuclear clustering between treatment conditions. (F) U87-MG cells were treated with DMSO, 2 μM Y102, 50 μM HCQ or 100 µM tBHP, an inducer of oxidative stress, for 24 h. At 30 min prior to collection, cells were stained with MitoTracker Red CMXROS (red). Cells were stained for oxidative stress marker 8-hydroxyguanosine (8-OHG) (green). DAPI was used to counterstain cell nuclei (blue). Scale bar: 50 µm. (G) Quantification of 8-OHG positive cells as in F. 100 cells were analyzed in triplicate experiments. N.S., not significant (*P*>0.1234);  **P<0.0021; ***P<0.0002; ****P<0.0001 (one-way ANOVA with multiple comparisons using the Dunnett correction method). Results in B,E,G are the mean±s.d.

Finally, we examined the impact of Y102 on autophagic processes by surveying expression of the adaptor protein sequestosome-1 (p62; also known as SQSTM1), a shared marker of autophagy and oxidative stress perturbations (Liu et al., 2016; Song et al., 2017). p62 recognizes and binds to ubiquitin on cargo marked for degradation either by autophagy or the proteasome; furthermore, phosphorylation of NF-κB following oxidative stress induction results in the upregulation of p62 protein (Nedelsky et al., 2008; Song et al., 2017). Y102 increased expression of p62 compared to DMSO (Fig. 2A,B). We also noted that p62 expression with Y102 treatment was similar to that previously observed using the late-stage autophagy inhibitor hydroxychloroquine (HCQ) (Mauthe et al., 2018). We also examined the effect of Y102 on the lipidation state of microtubule–associated protein light chain 3 (LC3) family proteins, another autophagy marker (Tanida et al., 2008). Cytosolic (non-lipidated) LC3-I is converted into LC3-II via phosphatidylethanolamine addition, and LC3-II is recruited to autophagosome membranes (Shibutani and Yoshimori, 2014). When autophagy is inhibited at a stage following the lipidation of LC3-I with compounds like HCQ, accumulation of LC3-II is detectable; however, when autophagy is stimulated and unhindered, LC3-II is degraded within the autolysosome and only LC3-I is detectable (Mizushima and Yoshimori, 2007). Based on this knowledge, we hypothesized that LC3-II would accumulate after Y102 treatment if it inhibits macroautophagy. To test this, we treated U87-MG cells with Y102 for 12 h, followed by HCQ co-treatment for an additional 12 h, for a total of 24-h treatment (Fig. 2C). We found that, although p62 expression increased with Y102 or HCQ treatment, Y102 treatment alone did not result in LC3-II accumulation. All treatments with HCQ present resulted in LC3-II accumulation, suggesting that, although HCQ and Y102 both increase expression of p62, the mechanisms of action of Y102 and HCQ differ, and Y102 is unlikely to be a macroautophagy inhibitor.

Previous studies have shown that tumors with dysregulated RAS, which include *NF1*-deficient tumors, depend on autophagy to modulate accumulation of oxidative stress-induced damaged mitochondria, and defective autophagic clearance results in the accumulation of abnormal mitochondria (Guo et al., 2011). It has also been previously demonstrated that loss of neurofibromin results in a decrease in expression and activity of key components of oxidative phosphorylation. Furthermore, *NF1*-deficient cells have been reported to have a significant increase in reactive oxygen species (ROS) (Bessler et al., 2016; Masgras et al., 2022), suggesting that *NF1*-deficient cells will require greater mitochondrial turnover than their wild-type counterparts. To investigate whether Y102 resulted in an abnormal mitochondrial phenotype, we used the membrane potential-based fluorescent probe MitoTracker Red CMXRos. Whereas cells treated with DMSO had a highly structured network of mitochondria, Y102-treated cells exhibited perinuclear clustering of the mitochondria, something not observed with HCQ (Fig. 2D,E).

To investigate whether Y102 resulted in an increase in oxidative damage in *NF1*-deficient cells, we probed for 8-hydroxyguanosine (8-OHG), an oxidative stress-induced DNA damage marker for both nuclear and mitochondrial DNA (Song et al., 2017; Valavanidis et al., 2009). Y102 resulted in an increase in 8-OHG compared to DMSO, suggesting an increase in oxidative stress and subsequent damage following Y102 treatment (Fig. 2F,G). Localization of 8-OHG indicated that the oxidative stress-induced damage affected both nuclear and mitochondrial DNA, as 8-OHG expression colocalized in both the mitochondria in the cytoplasm and the nucleus. Together, these findings suggest a potential mechanism of action of Y102, whereby it results in a defect to clear mitochondria by an autophagic process.

### Y102 alters localization of the mitophagy receptor BNIP3L

**Fig. 3.**
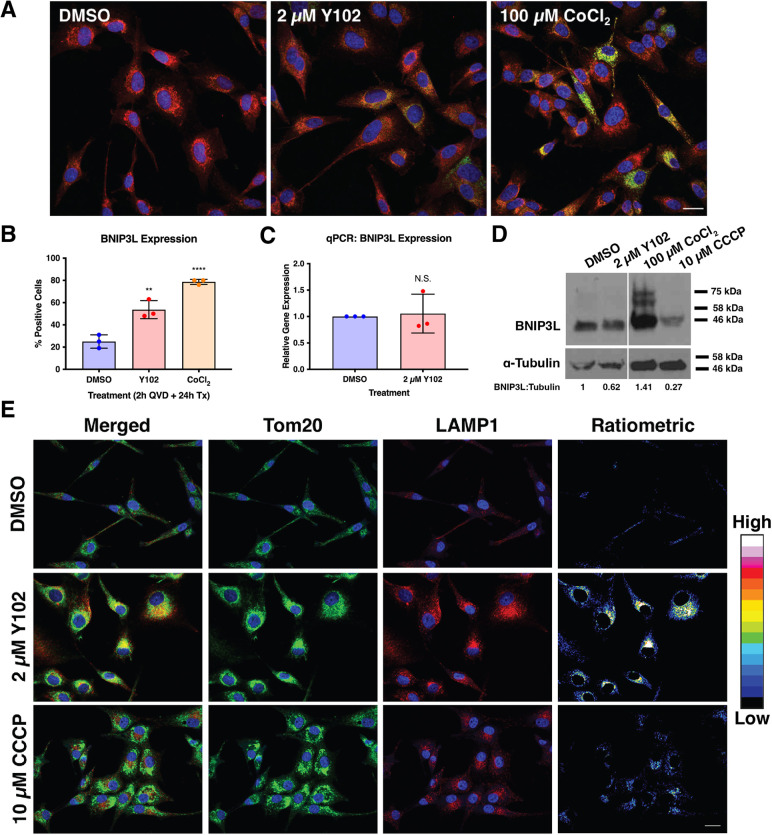
**Treatment with Y102 prevents lysosome-mediated mitochondrial clearance.** (A) U87-MG cells were treated for 24 h with DMSO, 2 μM Y102 or 100 μM CoCl_2_. 30 min prior to collection, cells were stained with MitoTracker Red CMXROS (red). Cells were also stained for the mitophagy receptor BNIP3L (green). DAPI was used to counterstain cell nuclei (blue). Scale bar: 50 µm. (B) Quantification of BNIP3L expression. 100 cells were analyzed from triplicate experiments. (C) RT-qPCR analysis of *BNIP3L* mRNA expression following Y102 treatment compared to DMSO from triplicate experiments. (D) Western blotting analysis of BNIP3L expression after 2 h of 20 μM Q-VD-OPh hydrate (QVD) pre-treatment, followed by 24 h co-treatment with DMSO, 2 μM Y102, 100 μM CoCl_2_ or 10 μM CCCP. α-Tubulin served as a loading control. Vertical white line indicates where image was spliced; samples shown are all from the same blot. Densitometry analysis was used to determine the ratios of BNIP3L to tubulin. Values are for blot shown. Blot is representative of three experiments. (E) U87-MG cells were treated for 16 h with DMSO, 2 μM Y102, 2 μM JW-1, 50 μM HCQ or 100 μM CoCl_2_. Cells were stained for the lysosome marker LAMP1 (red) and the mitochondria marker Tom20 (green). DAPI was used to counterstain cell nuclei (blue). Ratiometric images comparing the colocalization between mitochondria and lysosomes were generated using Fiji. Resulting fluorescence is displayed as intensities (16-color; color bar on right). Images are representative of three repeats. Scale bar: 50 µm. N.S., not significant (*P*>0.1234); **P<0.0021; ****P<0.0001 (one-way ANOVA with multiple comparisons using the Dunnett correction method). Results in B,C are the mean±s.d.

It is known that there are a multitude of selective forms of autophagy aside from non-selective macroautophagy (Mancias and Kimmelman, 2016). Although p62 can play a role in some forms of mitochondrial degradation via autophagy (known as mitophagy), it can also be involved in a host of other selective autophagy processes, as well as non-selective autophagy (Mancias and Kimmelman, 2016). We therefore decided to determine whether Y102 resulted in an effect on mitophagy by examining expression of a mitophagy-specific receptor, BNIP3L (also known as Nix) (Hamacher-Brady and Brady, 2016). BNIP3-mediated mitophagy is reported to occur following perinuclear clustering and fragmentation of the mitochondria (Al-Mehdi et al., 2012). With Y102 treatment, we observed an increase in expression of BNIP3L on the mitochondria compared to that for the DMSO control (Fig. 3A,B). This localization of BNIP3L with Y102 was similar to that observed after treatment with the mitophagy inducer CoCl_2_. To address whether Y102 resulted in increased expression of BNIP3L or only a change in its localization, we analyzed protein expression levels using western blotting. There was little difference in BNIP3L protein levels between Y102-treated and DMSO-treated cells, suggesting that there was no increase in BNIP3L protein expression with Y102 but rather a change in its localization (Fig. 3C). To confirm this, we performed RT-qPCR to examine *BNIP3L* transcript levels. Again, there was little difference in *BNIP3L* RNA levels between DMSO and Y102 treatment, suggesting that Y102 resulted in a change in the localization of BNIP3L to the mitochondria (Fig. 3D).

### Y102 prevents lysosomal-directed mitochondrial clearance

Further exploration into the mechanism of Y102-mediated cell death led us to investigate late stages of mitochondrial clearance when the mitochondria-selective autophagosomes fuse with acidic lysosomes to form the autolysosome. An increase in lysosome expression was observed following Y102 treatment, and these lysosomes were found to localize in the perinuclear region of the cell (Fig. 3E). Colocalization between lysosomes and mitochondria was observed with Y102, as determined by ratiometric analysis, where the observance of yellow fluorescence is indicative of a colocalization between red and green fluorescence (Fig. 3E). Given that Y102-treated cells are unable to traffic mitochondria-containing lysosomes out of the cell periphery, as evidenced by the perinuclear clustering observed, it suggests that the mechanism of Y102-mediated cell death is in part due to a defect in mitophagy-driven mitochondrial clearance.

### Identification of the cellular targets of Y102 by click-chemistry-aided mass spectrometry and cellular thermal shift assay

**Fig. 4.**
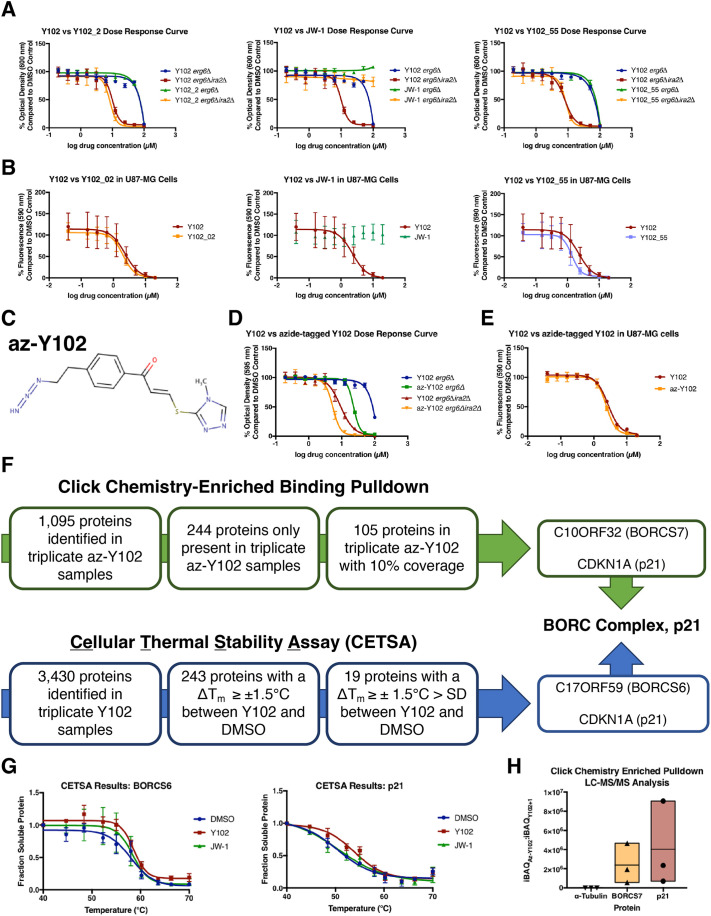
**Identification of potential targets of Y102 using a multipronged proteomics approach.** (A) Yeast were grown at 30°C starting at an OD_600_ of 0.05 with Y102 or various analogs of Y102 at concentrations ranging from 100 µM to 0.039 μM. At 18 h, OD_600_ was measured. (B) U87-MG cells were treated for 72 h with Y102 or various analogs of Y102 at concentrations ranging from 100 µM to 0.039 μM. At 3 h prior to collection, cells were stained with alamarBlue. (C) Chemical structure of azide-tagged Y102 (az-Y102), modification based on the structure–activity relationship studies performed with analogs of Y102. (D) Yeast were grown at 30°C starting at an OD_600_ of 0.05 with Y102 or az-Y102 at concentrations ranging from 100 µM to 0.039 μM. At 18 h, the OD_600_ was measured. (E) U87-MG cells were treated for 72 h with Y102 or az-Y102 at concentrations ranging from 100 µM to 0.039 μM. At 3 h prior to collection, cells were stained with alamarBlue. (F) Comparison between the two proteomics approaches, along with the implementation of additional criteria to the results, led to the identification of two potential targets of Y102: p21 and BORC. (G) CETSA results for p21 and BORCS6. Graphs represent triplicate analyses and measure the fraction of soluble protein following exposure to the indicated temperatures. (H) Click-chemistry enriched pulldown results for p21 and BORCS7. Graphs represent triplicate analyzes and measure the iBAQ area ratio between az-Y102 and parent compound Y102. α-Tubulin is included as a comparative control. Results in A, B, D and E are the mean±s.d. of four technical replicates. Results in G are the mean±s.d. of three experiments. For H, the box represents the range, and the median is indicated.

We observed that the effects of Y102 were irreversible after a 2 h treatment and hypothesized that this irreversibility could be leveraged to identify targets of Y102 using a click chemistry-enriched proteomics approach (Xu et al., 2016). For this we first carried out structure-activity relationship studies for Y102 to identify the key structural components of the compound required for its activity, with the ultimate goal of identifying a site on Y102 that could be modified with an azide group. We designed a total of 20 analogs of Y102, most of which had relatively little effect on the efficacy of the analog in both yeast and mammalian platforms, although some modifications resulted in complete loss of activity (Fig. 4A,B; Table S1, Fig. S3). Using our findings from these structure–activity relationship studies, we designed an azide-tagged version of Y102 to use in a click-chemistry-enhanced pulldown of proteins that bound Y102 (az-Y102; Fig. 4C). *IRA2*-deficient yeast were found to be preferentially sensitive to az-Y102, as was observed with the parent compound Y102, and a similar efficacy between Y102 and az-Y102 was observed in the U87-MG human tumor cell line model, making it a comparable analog to use in our target identification strategy (Fig. 4D,E).

U87-MG cells were treated with vehicle DMSO, the parent compound Y102 or az-Y102 for 2 h; then lysates were prepared and incubated with a resin-bound alkyne in the presence of copper sulfate to covalently pulldown all proteins bound to az-Y102. These proteins were digested using trypsin, and the peptides were eluted. Utilizing liquid chromatography-tandem mass spectrometry (LC-MS/MS), the peptide intensities and observable peptides of a protein were measured, and the ratios were compared between az-Y102 and Y102 (no azide tag) and DMSO controls. Using this procedure, we identified 105 proteins present in all az-Y102 samples in triplicate experiments, but not identified in the controls, with >10% of the protein identified in the peptide fragments analyzed (Table S2).

Simultaneously, we used a second, but separate, proteomics approach known as a cellular thermal shift assay (CETSA) to identify potential targets of Y102 by comparing the thermal stability proteome profiles between Y102-treated cells and control-treated cells (Jafari et al., 2014). Comparison of the proteome-wide thermal stability profiles identified 19 proteins with a statistically significant difference (*P*≤0.1) of their melting points between Y102 and vehicle control (ΔT_m_; Fig. 4F; Table S2). Upon comparison between the two proteomics approaches, two potential targets were identified: BLOC-one-related complex (BORC), as shown by the identification of two subunits BORCS6 and BORCS7, and the cell cycle regulator p21 (also known as CDKN1A) (Fig. 4G,H).

### BORC is a potential vulnerability in NF1-deficient cells

BORCS6 is part of the multiprotein BORC, which comprises eight proteins (Pu et al., 2015). BORC is associated with the cytosolic side of lysosomes and is required for the transport of lysosomes from the perinuclear region to the cell periphery along microtubules through anterograde transport. Lysosomes play important roles outside of their function as degradative organelles, including functions in plasma membrane repair, cell adhesion and migration, tumor invasion and metastasis, gene regulation and metabolic signaling (Pu et al., 2016). Loss of various BORC subunits is reported to result in perinuclear clustering of LAMP-1-positive lysosomes, similar to what we have observed in *NF1-*deficient cells in the presence of Y102 (Pu et al., 2015). Furthermore, trafficking of lysosomes to the cell periphery promotes cell transcription, translation and metabolic processes associated with proliferation (Pu et al., 2017).

**Fig. 5.**
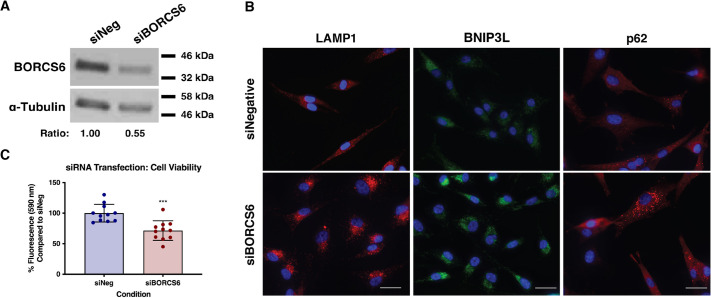
**Knockdown of a BORC subunit recapitulates the phenotypes of Y102 treatment.** (A) A western blot of lysates from U87-MG cells treated for 72 h with negative control siRNA (siNeg) or siRNA against BORCS6 (siBORCS6) was probed for BORCS6. Densitometry analysis was used to determine the ratio of BORCS6 to α-Tubulin control to determine sufficient knockdown. Densitometry analysis was used to determine their ratio for the blot shown. Blot is representative of two experiments. (B) U87-MG cells were treated with negative control siRNA (siNegative) or siBORCS6. Following 72 h knockdown, cells were stained for the lysosome marker LAMP1 (red), the mitophagy receptor BNIP3L (green), and the autophagy marker p62 (red). DAPI was used to counterstain cell nuclei (blue). Scale bars: 150 µm. Images are representative of three repeats. (C) U87-MG cells were treated for 72 h with siNeg or siBORCS6. At 3 h prior to collection, cells were stained with alamarBlue. Results are the mean±s.d. (*n*=3). ***P<0.0003 (unpaired two-tail *t*-test).

We therefore hypothesized that prevention of lysosome trafficking to the cell periphery through inhibition of BORC in *NF1*-deficient cells could recapitulate the phenotypes observed after Y102 treatment. To test this, we knocked down one subunit of BORC, BORCS6, using siRNA (Fig. 5A). Knockdown of BORCS6 resulted in perinuclear clustering of the lysosomes and an increase in p62 puncta as previously reported (Jia et al., 2017), recapitulating the phenotypes observed with Y102 (Fig. 5B, and see below). BORCS6 knockdown also resulted in a similar alteration in BNIP3L expression to that observed with Y102 (Fig. 5B). BORCS6 siRNA resulted in decreased viability of *NF1*-deficient cells compared to what was seen with control siRNA, similar to that observed with Y102 treatment (Fig. 5C).

### Knockdown of a BORC subunit or treatment with Y102 leads to increased p21 expression and nuclear size

**Fig. 6.**
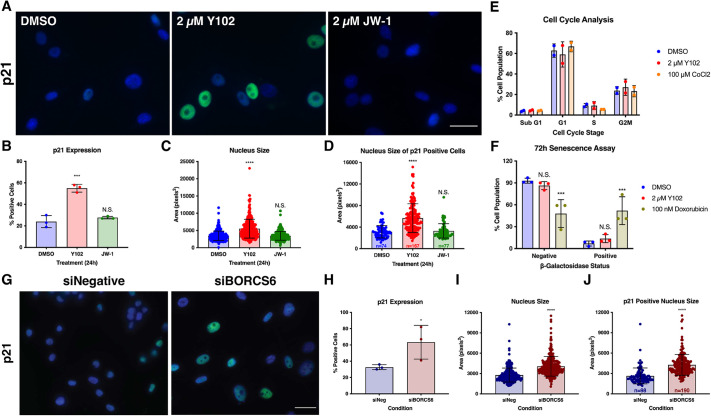
**Knockdown of a BORC subunit or treatment with Y102 leads to increased p21 expression and nuclear size.** (A) U87-MG cells were treated for 24 h with DMSO, 2 μM Y102 or 2 μM JW-1. Cells were stained for p21, a regulator of the cell cycle (green). DAPI was used to counterstain cell nuclei (blue). Scale bar: 150 µm. (B) Quantification of p21-positive cells for cells treated as per A. 100 cells were analyzed in triplicate experiments. (C) 100 cells were analyzed in triplicate experiments to determine the average nucleus size for cells treated as per A. (D) 100 cells were analyzed in triplicate experiments to determine the average nucleus size of p21-positive cells for cells treated as per A. Number of positive cells out of 300 total cells is indicated by n. (E) U87-MG cells were treated for 24 h with DMSO, 2 µM Y102 or 100 µM CoCl_2_. Following treatment, cells were trypsinized, permeabilized, and stained with DAPI. Data presented is the percentage of cells in each stage of the cell cycle as measured by flow cytometry for duplicate experiments. (F) To measure senescence, U87-MG cells were treated for 72 h with DMSO, 2 µM Y102 or 100 nM Doxorubicin. Following treatment, cells were fixed and stained with 1 mg/ml β-galactosidase solution. 100 cells were analyzed in triplicate to determine the number of β-galactosidase positive cells. (G) U87-MG cells were treated with negative (siNeg) or BORCS6-specific (siBORCS6) siRNA. Following 72 h knockdown, cells were stained for the cell cycle regulator p21 (green). DAPI was used to counterstain cell nuclei (blue). Scale bar: 150 µm. (H) Quantification of p21-positive cells. 100 cells were analyzed in triplicate experiments for cells treated as per G. (I) 100 cells in triplicate experiments were analyzed to determine the average nucleus size for cells treated as per G. (J) 100 cells in triplicate experiments were analyzed to determine the mean nucleus size of p21-positive cells for cells treated as per G. Results in B–F, H–J are the mean±s.d. N.S., not significant (*P*>0.1234), *P<0.0332, ***P<0.0002; ****P<0.0001 (one-way ANOVA with multiple comparisons using the Dunnett correction method).

Given that we identified p21 in our proteomics approaches as a potential target of Y102, we examined its expression following Y102 treatment. p21 is a cell cycle regulator, binding to and inhibiting cyclin-dependent kinases (CDKs) (CDK4 and CDK6 with cyclin-D, CDK2 and CDK1 with cyclin-E and cyclin-A, and CDK1 with cyclin-B) resulting in cell cycle arrest (Jung et al., 2010). Furthermore, p21 expression can mediate cellular senescence through a ROS-based mechanism, which can lead to flattening of the cell, which is reflected in an increase in nuclear size and a decrease in DNA content as measured by DAPI staining (Zhao and Darzynkiewicz, 2013). To determine whether p21 expression or nucleus size was altered with Y102 treatment, we analyzed both via immunofluorescence. We found that Y102 resulted in increases in both p21 expression and nucleus size, which correlated with each other (Fig. 6A–D). As p21 plays a role in cell cycle regulation and cellular senescence, we examined the cell cycle distribution, as indicated by DNA content, and senescence using a senescence-associated β-galactosidase assay of cells following Y102 treatment. In both instances, we found that whereas Y102 resulted in an increase in p21 expression there was no indication of cell cycle arrest or senescence (Fig. 6E,F). We also observed an increase in p21 expression and nucleus size following BORCS6 siRNA knockdown (Fig. 5A, Fig. 6G–J), suggesting that alterations in p21 expression might be a consequence of inhibition of BORC.

### BORC interacts with Y102

**Fig. 7.**
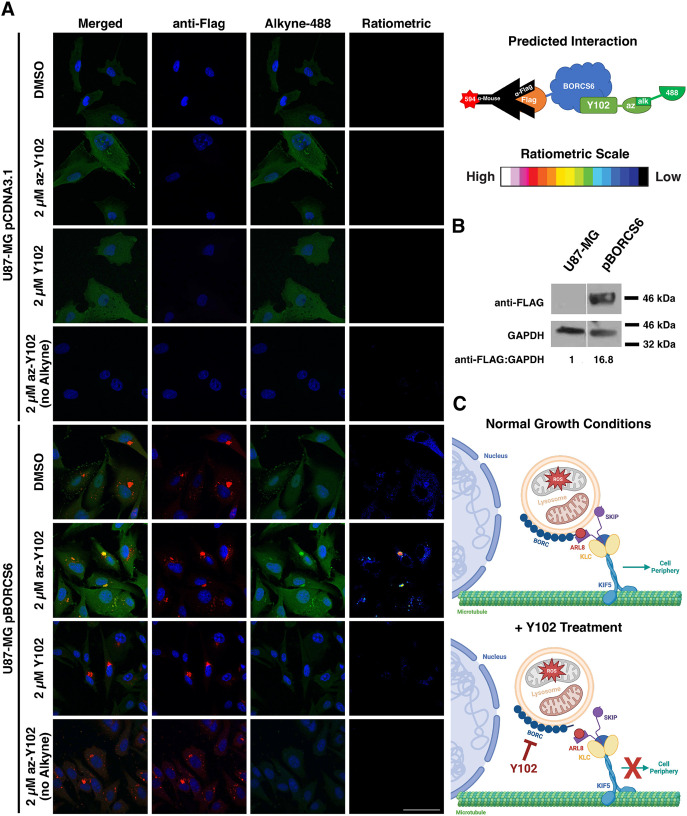
**BORC interacts with Y102.** (A) U87-MG cells containing an empty vector or expressing FLAG-tagged BORCS6 were treated with az-Y102, Y102 or DMSO. Afterwards, az-Y102 was labeled with alkyne-488 via click chemistry (green), and BORCS6 was visualized using anti-FLAG (red). Nuclei are labeled in blue. Ratiometric images comparing the colocalization between az-Y102 and BORCS6 were generated using Fiji software. Resulting fluorescence is displayed as intensities (16-color; color bar on right). Scale bar: 50 µm. Images are representative of two repeats. (B) A western blot of lysates from U87-MG cells or a clone expressing FLAG-tagged BORCS6 was probed for BORCS6. Densitometry analysis was used to determine the ratios of BORCS6 to GAPDH control to determine sufficient expression for blot shown. Blot is representative of two repeats. Vertical white line indicates where image was spliced; samples shown are all from the same blot. (C) Proposed mechanism of action for Y102. Lysosomal trafficking is carried out through the recruitment of Arl8 by BORC to the lysosome. Arl8 in turn recruits SKIP, which allows for the binding of KLC and kinesin 5. Kinesin 5 traffics the lysosome from the perinuclear region to the cell periphery. Y102 treatment prevents BORC-driven lysosomal trafficking, resulting in the perinuclear clustering of lysosomes.

To confirm the interaction between Y102 and BORCS6, we overexpressed FLAG-tagged BORCS6 or empty vector (EV) in U87-MG cells and treated cells with az-Y102, Y102 or DMSO for 2 h, following removal of the drug and incubation in medium without vehicle or drugs for an additional 22 h. az-Y102 was labeled with alkyne-488 using click chemistry, whereas overexpressed BORCS6 was detected with anti-FLAG. Confocal microscopy revealed that az-Y102 and BORCS6 colocalized in the cell, demonstrating that the cellular BORCS6 complexes are bound by Y102 (Fig. 7A). Overexpression was confirmed via western blotting (Fig. 7B). These findings suggest a potential mechanism of action of Y102 via inhibition of BORC (Fig. 7C).

## DISCUSSION

### Mechanism for Y102-mediated cell death

The work presented here demonstrates that Y102 alters lysosome positioning and impacts mitochondrial clearance in *NF1*-deficient cancer cells and is synthetic lethal with *NF1* loss in an isogenic yeast model. Additionally, the selectivity of Y102 for *NF1*-deficient yeast cells is recapitulated in an isogenic pair of *NF1*-deficient cell lines, as well as matched tumor and normal Schwann cell lines from an individual with NF1, demonstrating preferential sensitivity in cells that lack both copies of the *NF1* gene compared to wild-type or *NF1* heterozygous cells. This supports our hypothesis that the compound Y102 is selective in targeting *NF1*-deficient cells. We also found that sensitivity to Y102 has irreversible effects on *NF1-*deficient cell viability in as little as 2 h, demonstrating that the potential therapeutic effects of Y102 do not require continual drug presence. Furthermore, proteomics approaches and immunofluorescence imaging support that BORC is a target of Y102. Our working model is that Y102 prevents lysosome-directed mitochondrial clearance by preventing the normal function of BORC. This results in the perinuclear clustering of the lysosomes, as was observed with both Y102 treatment and knockdown of one subunit of BORC.

RAS dysregulated cancer cells rely on a high turnover of mitochondria due to their susceptibility to oxidative stress-induced damage; likewise, *NF1-*deficient tumor cells with RAS dysregulation might also require rapid mitochondrial turnover. Although autophagy is utilized as a survival mechanism for some tumors confronted with nutrient starvation and increased hypoxia, it might also play a tumor suppressor role by eliminating accumulation of p62 (Mathew et al., 2009). In the case of *NF1-*deficient cells, an increase in autophagic proteins, including p62, has previously been observed (Li et al., 2021). Conversely, a decrease in p62 expression is seen when NF1 is overexpressed, suggesting that *NF1-*deficient cells are more susceptible to autophagic modulation (Li et al., 2021). Indeed, in an *NF1*-deficient *Drosophila* cell line model, autophagic flux is increased compared to that in an isogenic control line; additionally, these cells are preferentially sensitive to the known late-stage autophagy inhibitors chloroquine and bafilomycin A1 (Stevens et al., 2024). Studies conducted in *NF1*-deficient murine cells show, in comparison to wild-type controls, downregulated respiratory complex I protein levels and enzymatic activity, leading to decreased respiration, a reduction in NAD^+^ and an increase in ROS (Masgras et al., 2022). Our study is consistent with these findings, as we observe an increase in oxidative stress specific to mitochondria in the presence of Y102. We also observed an inhibition of mitochondrial clearance with Y102 treatment, which leads to an accumulation of mitophagy-specific receptors and ROS-damaged mitochondria. Together, these findings suggest that inhibition of mitochondrial clearance via autophagy or prevention of lysosome distribution might be therapeutic strategies worth investigating further in the context of *NF1*-deficient tumors.

### Alterations of p21 expression with Y102 treatment and BORC knockdown

We identified two potential targets of Y102 using our dual proteomics approach: p21 and BORC. An increase in p21 expression was observed with both Y102 treatment and direct knockdown of a BORC subunit; however, there was no effect on cell cycle progression which is mediated by p21 expression. Furthermore, there was no indication of Y102-induced senescence despite this observed p21 increase. This raises the question as to why we detected elevated levels of p21 in both Y102-treated cells and cells transfected with siRNA against BORCS6. Increase in p21 expression both at the RNA and protein level following treatment with autophagic-lysosomal inhibitors has been reported (Qiao et al., 2013). Therefore, the observed p21 increase following Y102 treatment and BORCS6 knockdown might be due to inhibition of autophagic lysosome inhibition. The elevated p21 levels could explain the identification of p21 in both of our proteomic approaches. Alternatively, upregulation of p21 expression is reported to induce autophagy and to drive the onset of mitophagy, as well as mitochondrial dysfunction, in *NF1*-deficienct breast cancer cells (Capparelli et al., 2012). This suggests that increased p21 expression might prime *NF1*-deficient cells to be more susceptible to autophagy inhibition and, consequentially, Y102 inhibition.

### BORC as a potential vulnerability in NF1-deficient tumors

In addition to identifying a mechanism of action of Y102, this study is one of the first to suggest BORC as a potential therapeutic target in the context of cancer and tumorigenesis, and, to our knowledge, the first study to describe a small molecule capable of inhibiting BORC. BORC is responsible for lysosomal positioning in the cell, and knockdown of various subunits of BORC have led to perinuclear clustering of lysosomes, alterations in autophagic processes, and altered cell migration (Jia et al., 2017; Pu et al., 2015; Tunganuntarat et al., 2023). In yeast, the functions of BORC have been proposed to be carried out by BLOC1, which in mammals shares subunits with BORC (John Peter et al., 2013; Langemeyer and Ungermann, 2015). Trafficking of lysosomes to the cell periphery promotes cell transcription, translation and metabolic processes associated with proliferation; furthermore, lysosomal trafficking is reported to be regulated by mTORC1 signaling, a known vulnerability of *NF1*-deficient cells that is also the proposed mechanism of regulating autophagic activity (Li et al., 2021; Pu et al., 2017; Varin et al., 2016). Additional work in *Caenorhabditis elegans* has shown specifically that mTORC1 activation releases BORC and allows it to recruit the proteins necessary for lysosomal trafficking, like kinesin 1, as well as activation of Arl8 (Fazeli et al., 2023). In the context of breast cancer, high expression of *ARL8B* or BORC subunit genes is associated with poor prognosis, and expression of these proteins promotes invasion of cells that evade radiation treatment (Wu et al., 2020). Here, we show that knockdown of the BORCS6 subunit recapitulates the phenotypes observed following Y102 treatment, including accumulation of macroautophagy- and mitophagy-specific receptors, an increase in p21 expression and nucleus size, the perinuclear clustering of lysosomes and decreased viability of *NF1*-deficient cells. Together, the data presented in this study suggest that BORCS6 is a target of Y102, and inhibition of BORC is a potential vulnerability of *NF1*-deficient tumors. *NF1* deficiency leads to oncogenic RAS, MEK and ERK signaling (Ratner and Miller, 2015). Additionally, tumors with *NF1* loss share a pan-cancer RAS pathway activation RNA signature with tumors that have mutations in RAS family genes, including melanomas with *BRAF* mutations (Way et al., 2018). It is possible, therefore, that other tumors with oncogenic levels of RAS signaling will be susceptible to inhibition of BORC, which we are currently exploring.

## MATERIALS AND METHODS

### Reagents

Y102 was originally purchased from Maybridge (NRB04162SC). Subsequent stocks of Y102, along with JW-1, all Y102 analogs, and the azide-tagged form of Y102 were synthesized by Enamine (Kiev, Ukraine). Bortezomib (Bz; S1013), MG-132 (S2619), and Rapamycin (S1039) were purchased from Selleckchem. Hydroxychloroquine sulfate (HCQ; H1126) was purchased from Spectrum Chemical. tert-Butyl hydroperoxide solution (tBHP; 458139), carbonyl cyanide 3-chlorophenylhydrazone (CCCP; C2759) and hydroxyurea (HU; H8627) were purchased from Sigma-Aldrich. Staurosporine (STS; S-9300) was purchased from LC Laboratories. Cobalt (II) chloride (CoCl_2_; 36554) was purchased from Alfa Aesar. Q-VD-OPh hydrate (QVD; 50-101-3174) was purchased from Thermo Fisher Scientific. Stock solutions of compounds were prepared in 100% DMSO (Alfa Aesar) apart from HCQ and CoCl_2_, which were prepared fresh in DMEM (Corning Life Sciences) plus 10% fetal bovine serum (FBS) (Life Technologies).

### Cell culture

U87-MG, U251-MG, and sNF96.2 cells were purchased from ATCC. ipnNF95.11C and ipNF95.11b ‘C’ cells were kindly provided by Peggy Wallace, University of Florida, USA and are available for purchase through the ATCC. Immortalized mammary epithelial cells (IMECs) were a gift from James DiRenzo, Euroleader, Houston, TX, USA. U87-MG, U251-MG, ipnNF95.11C and ipNF95.11b ‘C’ cell lines were cultured in DMEM with L-glutamine, 4.5 g/l glucose and sodium pyruvate (Corning Life Sciences) with the addition of 10% (v/v) FBS (Atlanta Biologicals, Life Technologies or Gibco, Thermo Fischer Scientific). sNF96.2 cells were cultured in DMEM with 4 mM L-glutamine, 4.5 g/l glucose, 1 mM sodium pyruvate and 1.5 g/l sodium bicarbonate (ATCC) with the addition of 10% (v/v) FBS. IMECs were cultured in DMEM/F12 (1:1) supplemented with 5% FBS, 2 mM glutamine (Gibco), 5 μg/ml insulin (Akron Biotech), 500 μg/ml hydrocortisone (MP Biomedical) and 10 ng/ml recombinant human epidermal growth factor (Promega). All cell lines were grown at 37°C in 5% CO_2_, passaged regularly using PBS and 0.25% trypsin (Corning), and routinely screened for mycoplasma contamination using the MycoProbe kit (R&D Systems).

Although the currently available U87-MG cell line from ATCC differs from those derived from the original tumor, it is considered to be a human glioblastoma cell line of unknown origin (Allen et al., 2016). In the context of this work, U87-MG cells serve as an NF1-deficient tumor cell line model, due to elevated proteasome-mediated degradation of the NF1 protein (McGillicuddy et al., 2009). U87-MG cells have been used a model of an NF1-deficient tumor cell line in multiple studies (Allaway et al., 2016, 2017; Daginakatte and Gutmann, 2007; McGillicuddy et al., 2009; See et al., 2012).

### Yeast dose–response curves

*S. cerevisiae* strains were plated at an optical density at 600 nm (OD_600_) of 0.05 in 96-well plates (Falcon) and grown in Synthetic Complete medium (recipe according to Cold Spring Harbor Protocols https://cshprotocols.cshlp.org/content/2016/11/pdb.rec090589.full?sid=65431fb9-2fc5-4685-bbe6-85343665fa93) containing compound starting at 100 µM, followed by 2-fold serial dilutions to generate a 10-point range of concentrations in quadruplicate; one column was treated with equivalent amounts of DMSO (≤1%) as a control. Yeast were incubated for 18 h, after which the optical density was read using a Spectramax M2 (Molecular Devices) at a wavelength of 600 nm and normalized to DMSO.

### Hoechst cell viability assays

Cells were plated at 2500 cells/well in 96-well plates and allowed to adhere overnight. In the case of ipnNF95.11C and ipNF95.11b ‘C’, cells were plated at 5000 cells/well in 96-well plates. Medium was removed and replaced with medium containing compound starting at 20 µM or 100 µM, followed by 2-fold serial dilutions to generate a 10-point range of concentrations in quadruplicate; one column was treated with equivalent amounts of DMSO (≤1%) as a control. Cells were incubated for the indicated timepoints. Upon collection, medium was removed, cells were rinsed with PBS, and stored at −80°C until all plates were collected. To process cells, plates were thawed with PBS, incubated with 1× saline-sodium citrate (SSC) buffer with 0.02% SDS for 1 h at 37°C, followed by staining with 1 µg/ml Hoechst 33258 (Pierce). Absorbance was read using a Spectramax M2 at an excitation of 355 nm and an emission of 460 nm, and absorbance was normalized to DMSO.

### Cell viability assays using alamarBlue

Cells were plated at 5000 cells/well in 96-well plates and allowed to adhere overnight. Medium was removed and replaced with medium containing compound starting at 20 µM or 100 µM, followed by 2-fold serial dilutions to generate a 10-point range of concentrations in quadruplicate; one column was treated with equivalent amounts of DMSO (≤1%) as a control. Cells were incubated for 72 h. At 3 h prior to the end of incubation, alamarBlue (Invitrogen) was added to a final concentration of 5% v/v. Fluorescence was read using a Spectramax M2 at an excitation of 544 nm and an emission of 590 nm, and fluorescence was normalized to DMSO.

### Generation of NF1-deficient IMEC lines using CRISPR/Cas9 gene editing

A single guide RNA (sgRNA) was utilized to disrupt *NF1* in exon 2: 5′-GTTGTGCTCAGTACTGACTT-3′ (Shalem et al., 2014). IMECs were plated at 900,000 cells per 10 cm plate and were allowed to adhere overnight. The next day, cells were transfected with lentiCRISPRv2 (#52961 Addgene) and gRNA at a 3:1 ratio using Lipofectamine 2000 (Invitrogen) following the manufacturer's protocol. Clones were selected following 3 days of puromycin selection and confirmed to have loss of NF1 protein by western blotting.

### Immunofluorescence staining

U87-MG cells were plated at a concentration of 50,000 cells/well onto pre-coated poly-D-lysine (PDL) coverslips (Neuvitro) in 24-well plates (Falcon) and allowed to adhere overnight. Medium was removed and replaced with treatment-laced medium normalized to DMSO, and cells were treated for the indicated timepoints. At the time of collection, cells were washed with PBS and fixed with 4% paraformaldehyde without methanol (EMS); in the case of anti-γ-H2AX, cells were fixed with 100% methanol (Thermo Fisher Scientific). Coverslips were washed with PBS containing 0.05% Tween 20 (PBST; Thermo Fisher Scientific). Following permeabilization with 0.5% Triton X-100 in PBS (Thermo Fisher Scientific), coverslips were blocked for 1 h at room temperature (RT) in IF buffer (PBS containing 0.05% azide, 0.2% Triton X-100 and 2% normal goat serum or, for 8-OHG, normal donkey serum). For anti-LAMP1 staining, an additional permeabilization step in 100% methanol was included prior to permeabilizing with Triton X-100. The following primary antibodies were used for 1 h at RT unless specified: Alexa Fluor 488 pre-conjugated anti-γ-H2AX (N1-431) mouse monoclonal (1:50 for 30 min; cat. no 560445, BD Biosciences), anti-cleaved caspase 3 (Asp175) rabbit polyclonal (1:200; cat. no 9661, Cell Signaling Technology), anti-SQSTM1/p62 (D-3) mouse monoclonal (1:500; cat. no sc-28359, Santa Cruz Biotechnology), anti-8-hydroxyguanosine goat polyclonal (1:200; cat. no DR1001, Millipore), anti-BNIP3L/Nix (D4R4B) rabbit monoclonal (1:200, overnight at 4°C; cat. no 12396, Cell Signaling Technology), anti-Tom20 (FL-145) rabbit polyclonal (1:200; cat. no sc-11415, Santa Cruz Biotechnology), anti-LAMP1 (D4O1S) mouse monoclonal (1:100, overnight at 4°C; cat. no 15665, Cell Signaling Technology), anti-FLAG (M2) mouse monoclonal (1:500 for 2 h at 37°C; cat. no F1804, Sigma-Aldrich). Following washes with PBST, cells were stained with goat anti-rabbit-IgG or goat anti-mouse-IgG conjugated to Alexa Fluor 488 or Alexa Fluor 594 (or, for 8-OHG, donkey anti-goat-IgG conjugated to Alexa Fluor 488) secondary antibody at 1:800 for 1 h at RT (Jackson ImmunoResearch). Coverslips were washed with PBST following secondary staining, cells were stained with 0.33 μg/ml DAPI in PBS and mounted onto slides in ProLong Gold (Life Technologies).

For MitoTracker Red CMXRos staining, 30 min prior to collection, medium was removed and replaced with medium containing MitoTracker Red CMXRos at a final concentration of 100 nM (Molecular Probes). Following staining, cells were washed with PBS and fixed with 4% paraformaldehyde without methanol. Cells were washed with PBST, then permeabilized with 0.5% Triton X-100 in PBS. When co-staining with an additional marker, the staining procedure continued with the blocking step; otherwise, cells were stained with 0.33 μg/ml DAPI in PBS and mounted onto slides in ProLong Gold (Life Technologies).

To visualize the localization of az-Y102 using immunofluorescence, cells were treated for 2 h with az-Y102, DMSO or parent compound Y102, medium was removed and replaced with normal medium, and cells were incubated for a total of 24 h. Following treatment, cells were washed, fixed with 4% paraformaldehyde without methanol (EMS), and permeabilized using 0.5% Triton X-100 in PBS. Samples were stained with alkyne-488 at a final concentration of 1 µM following the Click-iT cell reaction buffer kit (Invitrogen) protocol. Following washes with 2% BSA, the staining procedure continued with the blocking step as described above.

All confocal images were acquired on a Nikon A1RSi confocal microscope equipped with a 60×1.4 NA oil objective, a DU4 detector unit and Nikon Elements software. Otherwise, images were acquired on a Zeiss Axio Imager.Z1 equipped with a Zeiss EC Plan-NEOFLUAR 40×1.3 NA oil objective, a Zeiss AxioCam MRm camera, a Lumen Dynamics Series 120 Q X-Cite Fluorescence Illuminator, and AxioVision SE64 Rel. 4.9.1 software. Image processing was performed with Fiji, built on ImageJ2.

### MV-151 proteasome active site inhibition assay

U87-MG cells were plated at 500,000 cells/well in six-well plates and allowed to adhere overnight (Falcon). Medium was removed and replaced with fresh medium containing 2 µM, 4 µM or 10 µM Y102 or equivalent amounts of vehicle DMSO (≤1%) and treated for 24 h. As a positive control, one well was treated for 2 h with 1 µM Bz and 10 µM MG-132. Cells were collected after treatment by washing with PBS, harvested with trypsin plus agitation to detach cells, spun down in a centrifuge (400 ***g*** for 5 min), and resuspended in PBS to wash. Cells were transferred to 1.5 ml microcentrifuge tubes, pelleted (600 ***g*** for 5 min) and resuspended in 20 µl digitonin lysis buffer (50 mM Tris-HCl pH 7.5, 250 mM sucrose, 2 mM EDTA, 1 mM ATP, 1 mM DTT and 0.05% digitonin). Cells were mixed by pipetting, incubated on ice for 20 min, and the lysate was cleared by centrifugation (≥18,000 ***g*** for 20 min). Protein concentrations were determined using a Bradford protein assay (Bio-Rad). 10 µg of protein lysate was incubated for 20 min with 20 µM MV-151 in a 37°C water bath. 4× loading dye was added to the samples and proteins were separated by SDS-PAGE on a 4–15% polyacrylamide gradient gel (Bio-Rad). Gels were imaged on a Typhoon scanner at an excitation of 532 nm and an emission of 560 nm to detect MV-151 fluorescence. Following imaging, proteins were transferred to nitrocellulose. The blot was probed for α-tubulin as a loading control. Experiments were repeated twice, and a representative image is shown.

### Western blotting

U87-MG cells were plated at 500,000 cells/well in six-well plates and allowed to adhere overnight (Falcon). Medium was removed and replaced with fresh medium containing drugs or compounds for the indicated timepoints. Cells were collected after treatment by washing with PBS, harvested with trypsin plus agitation to detach cells, pelleted in a centrifuge (400 ***g*** for 5 min) and resuspended in PBS to wash. Cells were transferred to 1.5 ml microcentrifuge tubes, pelleted (600 ***g*** for 5 min), and resuspended in RIPA lysis buffer (50 mM Tris-HCl pH 8.0, 150 mM NaCl, 1% nonidet P40, 0.5% sodium deoxycholate, and 0.05% SDS) containing 1 mM NaVO_4_, 1 mM NaF, 1 mM PMSF, 0.1 μg/ml antipain, 1 μM aprotinin, 100 μM benzamidine HCl, 0.1 μg/ml leupeptin, 0.1 μg/ml pepstatin and 0.1 μg/ml soybean trypsin inhibitor. Lysates were sonicated (2 × 5 s), incubated on ice for 15 min, and the lysate was cleared by centrifugation (≥18,000 ***g*** for 20 min). Protein levels were quantified using a BCA assay kit (Pierce). 50 µg of protein was prepared in 1× Laemmli sample buffer [50 mM Tris-HCl pH 6.8, 0.02% (w/v) Bromophenol Blue, 2% (w/v) SDS, 10% (v/v) glycerol, 1% (v/v) β-mercaptoethanol and 12.5 mM EDTA] and separated by SDS-PAGE on a 4–15% polyacrylamide gradient gel (Bio-Rad). Western blots for NF1 and phospho(p)-ERK were performed as above except lysates were run using a 3–8% NuPAGE gel (Invitrogen). Protein was transferred to a nitrocellulose membrane, blocked with 5% nonfat dry milk in TBST and probed with anti-NF1 (D7R7D) rabbit monoclonal (cat. no 14623, Cell Signaling Technology, 1:1000, overnight at 4°C), anti-PARP (46D11) rabbit monoclonal (cat. no 9532, Cell Signaling Technology, 1:5000, overnight at 4°C), anti-BNIP3L/Nix (D4R4B) rabbit monoclonal (cat. no 12396, Cell Signaling Technology, 1:1000, overnight at 4°C), anti-p62/SQSTM-1 (D-3) mouse monoclonal (cat. no sc-28359, Santa Cruz Biotechnology, 1:1000, 1 h at RT), anti-LC3BI/II rabbit polyclonal (cat. no #2775, Cell Signaling, 1:1000, overnight at 4°C), anti-C17orf59/BORCS6 rabbit polyclonal (cat. no PA566346, Invitrogen, 1:1000, overnight at 4°C), anti-GAPDH (14C10) rabbit monoclonal (cat. no 2118, Cell Signaling Technology, 1:2500, 1 h at RT), or anti-α-tubulin (B-1-2-5) mouse monoclonal (cat. no sc-23948, Santa Cruz Biotehcnology, 1:10,000, 1 h at RT) primary antibody in 2% milk in TBST. Secondary labeling was performed with a 1-h incubation in 1:10,000 anti-rabbit-IgG conjugated to HRP or anti-mouse-IgG conjugated to HRP (Jackson Immunoresearch) antibody diluted in 2% milk in TBST. The film was exposed to ECL-coated blots (Pierce) and developed using a standard film processor. Blot transparency images can be viewed in Fig. S4.

### Flow cytometry

U87-MG cells were plated at 500,000 cells/well in a six-well plate and allowed to adhere overnight. The medium was replaced with cell culture medium containing equivalent amounts of DMSO (≤1%), 2 µM Y102, 100 nM doxorubicin or 100 µM CoCl_2_ for 24 h. At the end of the incubation, cells were rinsed twice with PBS, trypsinized, and rinsed again with PBS prior to fixation with BD cytofix/cytoperm for 30 min on ice. After washing with BD perm/wash twice, cells were stained with DAPI at a final concentration 0.33 µg/ml in PBS for 30 min on ice. Cells were washed twice more with BD perm/wash and resuspended in PBS for analysis. The cells were transferred to flow cytometry tubes (14-961-10, Fisherbrand) and analyzed using a MacsQuant VYB 8-color flow cytometer. DNA content was detected using the V1 channel. This experiment was repeated twice, and the percentage of cells in each cell cycle stage is shown. 50,000 events per sample were collected and cellular debris was gated out of the dataset.

### RT-qPCR to measure *BNIP3L* transcript levels

U87-MG cells were plated into six-well plates at 250,000 cells/well and allowed to adhere overnight. The next day, cells were treated for 6 h with Y102 or vehicle control; RNA was collected at the endpoint using the RNAeasy Mini Kit (Qiagen). A total of 1.5 µl of RNA was converted into cDNA using first-strand cDNA synthesis kit SuperScript II RT (Invitrogen) following the manufacturer's protocol. cDNA was prepared with SYBR Green qPCR master mix (Applied Biosystems) and qPCR was performed using primers against *BNIP3L* or the internal control 18S on a StepOnePlus Real-Time PCR System (Applied Biosystems) in triplicate. ΔΔ_Ct_ was used to calculate relative gene expression. The following qPCR primer sequences were used: *BNIP3L* forward, 5′-AATGTCGTCCCACCTAGTCG-3′; *BNIP3L* reverse 5′-TAGCTCCACCCAGGAACTGT-3′; 18S rRNA forward, 5′-ATACAGCCAGGTCCTAGCCA-3′; 18S rRNA reverse, 5′-AAGTGACGCAGCCCTCTATG-3′.

### Cellular thermal shift assay and TMT 10-plex labeling

U87-MG cells were plated into two 10 cm tissue culture dishes at a concentration that would result in a yield of ∼1.5 mg total protein per plate on the day of collection. Cells were treated with 2 µM Y102, 2 µM JW-1 or the equivalent amount of DMSO (≤1%) for 2 h. The medium was removed and collected in a conical tube; cells were rinsed with PBS and trypsinized. Cells were re-suspended in 1 ml PBS with protease inhibitors (Roche), equating to a protein concentration of ∼1 mg/ml. 100 µl of resuspended cells were transferred to PCR tubes and incubated for 3 min in thermal cyclers preheated to the following temperatures: 37, 37, 44.7, 48.4, 52.3, 55, 58, 60.2, 63.3, 66.3 and 70 (°C). Cells were lysed by freeze-thaw and centrifuged at 21,100 ***g*** for 20 min at 4°C. Supernatants were removed to a fresh 0.5 ml Eppendorf tube.

Supernatant from the duplicate 37°C treatment condition was used to determine protein concentration through a BCA assay. This allowed for the quantitative transfer of 50 µg of protein in each of the remaining tubes to a fresh 1.5 ml Eppendorf tube. Proteins were denatured with 7 M urea, reduced with DTT and alkylated with iodoacetamide. Urea was diluted to a concentration of <1 M with Tris-HCl pH 8.1 and proteins were digested with proteomics grade trypsin. Peptides were acidified and desalted via solid-phase extraction and evaporated by vacuum centrifugation. Each temperature treatment was differentially labeled using TMT-10-plex reagent (Thermo Fisher Scientific).

### TMT LC-MS/MS analyses

The Orbitrap Fusion was operated with an Orbitrap MS1 scan at 120K resolution and an AGC target value of 500K. The maximum injection time was 100 ms, the *m*/*z* range was 350 to 1300 and the dynamic exclusion window was 30 s. Precursor ions were selected for MS2 using quadrupole isolation (0.6 *m*/*z* isolation width) in a ‘top speed’ (3 s duty cycle), data-dependent manner. Ion charge states of +2 through +5 were selected for MS2 by collision induced dissociation (CID) fragmentation (32% CID energy) and ion trap analysis. The MS2 scan maximum injection time was 60 ms and AGC target value was 8K. MS2 fragment ions were selected for synchronous precursor selection (SPS)-MS3 analysis in a top 10 data-dependent manner. MS3 scans were generated through higher energy collision-induced dissociation (HCD) fragmentation (55% HCD energy) and Orbitrap analysis at 60K resolution, with a scan range of 110 to 750 *m*/*z*. The MS3 scan maximum injection time was 200 ms and AGC target value was 50K.

### Click chemistry sample TCA precipitation and LC-MS/MS analyses

4×10^7^ cells were plated into a total of 3–15 cm dishes per treatment condition and allowed to adhere overnight. Cells were treated for 2 h with the equivalent amount of DMSO (≤1%), 2 µM Y102 or 2 µM azide-tagged Y102. Following treatment, cells were washed with PBS, trypsinized and resuspended in 850 µl urea lysis buffer (8 M urea, 200 mM Tris-HCl pH 8, 4% CHAPS, 1 M NaCl) from the Click-iT Protein Enrichment Kit (Molecular Probes). Samples were incubated on ice for 10 min prior to sonication six times for 3 s each. Lysates were centrifuged at 10,000 ***g*** for 10 min, then 800 µl of the lysate was added to 200 µl of alkyne-bound resin slurry and 1 ml of catalyst solution containing copper (II) sulfate at a final concentration of 1 mM. Slurries were rotated end-over-end at RT for 18 h. Following this, samples were reduced using 1 M DTT and 7.4 mg/ml of iodoacetamide, stringently washed using SDS wash buffer, 8 M urea, and 20% acetonitrile, precipitated in 20% TCA, and washed in 10% TCA and cold acetone. Precipitated proteins were digested with trypsin and peptides identified by LC-MS/MS on an Orbitrap Fusion as described below.

### LC-MS/MS analyses of TCA precipitated material

LC-MS/MS analysis was performed on an Orbitrap Fusion Tribrid mass spectrometer (Thermo Fisher Scientific) equipped with an EASY-nLC 1000 ultra-high-pressure liquid chromatograph (Thermo Fisher Scientific). Peptides were dissolved in loading buffer [5% methanol (Fisher)/1.5% formic acid] and injected directly onto an in-house pulled polymer-coated fritless fused silica analytical resolving column (40 cm length, 100 µm inner diameter; PolyMicro) packed with ReproSil, C18 AQ 1.9 µm 120 Å pore (Dr. Maisch). Samples were separated with a 90-min gradient of 4 to 33% LC-MS buffer B (LC-MS buffer A: 0.125% formic acid, 3% ACN; LC-MS buffer B: 0.125% formic acid, 95% ACN) at a flow rate of 330 nl/min. The Orbitrap Fusion was operated with an Orbitrap MS1 scan at 120K resolution and an AGC target value of 500K. The maximum injection time was 100 ms, the scan range was 350 to 1500 *m*/*z* and the dynamic exclusion window was 15 s (±15 ppm from precursor ion *m*/*z*). Precursor ions were selected for MS2 using quadrupole isolation (0.7 *m*/*z* isolation width) in a ‘top speed’ (2 s duty cycle), data-dependent manner. MS2 scans were generated through HCD fragmentation (29% HCD energy) and Orbitrap analysis at 15 K resolution. Ion charge states of +2 through +4 were selected for HCD MS2. The MS2 scan maximum injection time was 60 ms and the AGC target value was 60 K.

### Peptide spectral matching and bioinformatics

Raw data were searched using COMET against a target-decoy version of the human (*Homo sapiens*) proteome sequence database (UniProt; downloaded 2013; 20,241 total proteins) with a precursor mass tolerance of ±1.00 Da and requiring fully tryptic peptides with up to three missed cleavages, carbamidomethyl cysteine as a fixed modification and oxidized methionine as a variable modification (Eng et al., 2013). For TMT experiments, the TMT reagent mass was searched as a static modification on lysine residues and peptide N-termini. The resulting peptide spectral matches were filtered to <1% false discovery rate (FDR) by defining thresholds of decoy hit frequencies at particular mass measurement accuracy (measured in parts-per-million from theoretical), XCorr and delta-XCorr (dCn) values. TPP-TR analyses were performed using the R statistical programming language (http://www.R-project.org) (Franken et al., 2015).

### siRNA knockdown

To knockdown BORCS6, cells were plated at 200,000 cells/well in six-well plates, 50,000 cells/well on precoated PDL coverslips in 24-well plates, or 5000 cells/well in 96-well plates and allowed to adhere overnight. The next day, cells were transfected with negative control siRNA (Ambicon; #AM4611) or siRNA targeting BORCS6 (Invitrogen; HSS123247) at a final concentration of 2 pmol/µl and lipofectamine 2000 (Invitrogen) in Opti-MEM medium (Gibco), and the mixture was incubated for 20 min at room temperature prior to drop-wise addition to cells. At 24 h after transfection, reagents were added to the culture, and medium was removed and replaced with normal growth medium. Cells were incubated for a total of 72 h before collection.

### β-galactosidase senescence assay

U87-MG cells were plated at 100,000 cells/well and allowed to adhere overnight. Medium was removed and replaced with medium containing equivalent amount of DMSO (≤1%), 2 µM Y102 or 100 nM doxorubicin for a total of 72 h. Cells were collected and stained for β-galactosidase using the Senescence β-Galactosidase Staining Kit (Cell Signaling). 100 cells were counted per condition; the graph represents results from triplicate experiments.

### Stable transfection of BORCS6 in U87-MG cells

To establish U87-MG cells overexpressing BORCS6, cells were plated at 100,000 cells/well in a 12-well plate and allowed to adhere overnight. The next day, cells were transfected with pcDNA3.1 empty vector or pBORCS6 (GenScript) at a final concentration of 8 ng/µl and Lipofectamine 2000 (Invitrogen) in Opti-MEM medium (Gibco), and the mixture was incubated for 20 min at RT prior to drop-wise addition to cells. At 24 h after transfection, reagents were added to the culture, and medium was removed and replaced with normal growth medium containing 1000 µM G418 (Gibco). After several days, pBORCS6 underwent clonal selection; expression of BORCS6 was confirmed by immunofluorescence.

## Supplementary Material

10.1242/joces.262343_sup1 Supplementary information

Table S2. Click-chemistry-aided mass spectrometry and CETSA results
