# Supplementary material for: Chemical genetic screens reveal defective lysosomal trafficking as synthetic lethal with NF1 loss
Source: J Cell Sci. 2024 Aug 14;137(15):jcs262343. doi: 10.1242/jcs.262343 (PMC11361638; doi:10.1242/jcs.262343)
Supplement: Supplementary information [file joces-137-262343-s1.pdf]

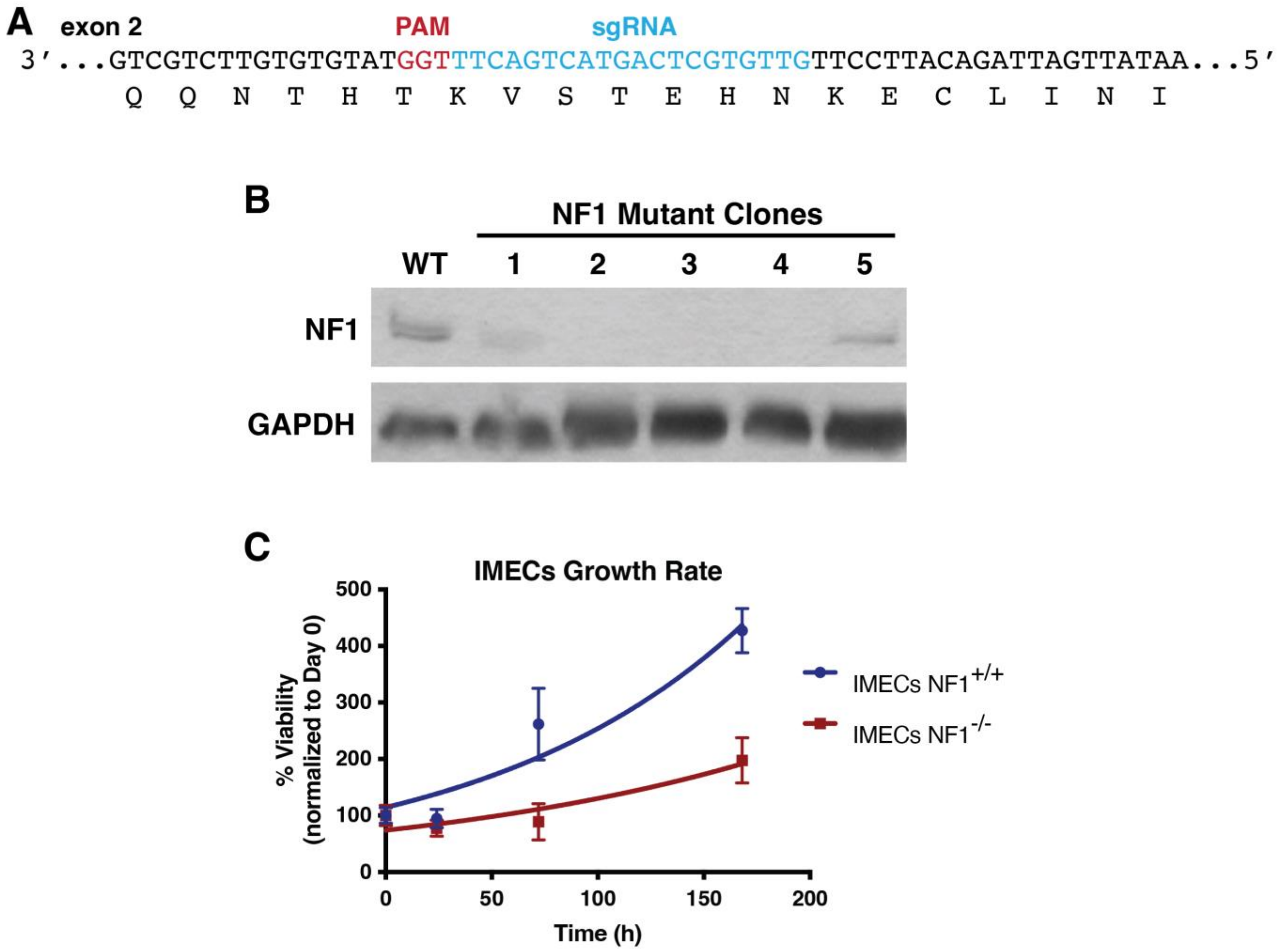

Fig. S1. Generation of NF1-deficient cell line using CRISPR/Cas9 gene editing

CRISPR/Cas9 was used to target exon 2 of *NF1* in a telomerase-immortalized human mammary epithelial cell line (IMECs) to generate *NF1* knockout (*NF1*$^{-/-}$) cell lines. (A) sgRNA and PAM site used along with wild type Cas9 to generate knock out of *NF1*. (B) Western blot analysis of multiple cell lines showing reduced and absent levels of neurofibromin compared to the wild type IMEC line. We utilized line 2 for subsequent experiments. GAPDH was used as a loading control. (C) Growth of WT (*NF1*$^{+/+}$) and *NF1* knockout mutant 2 IMEC (*NF1*$^{-/-}$) lines was measured by collecting 96-well plates at designated timepoints for up to 7 days. For collection, cells were stained with 1µg/mL Hoechst 33258 and absorbance was read at ex/em 355/460 nm. All measurements were normalized to a day 0 control.

Journal of Cell Science • Supplementary information

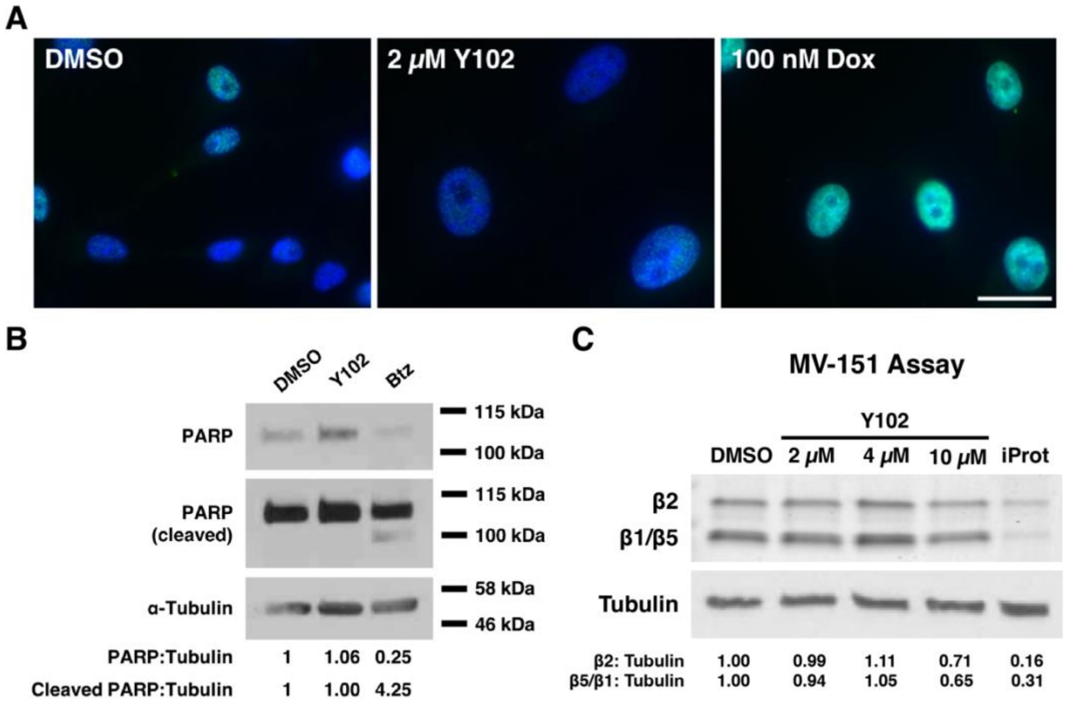

Fig. S2. The mechanism of Y102-mediated cell death is not driven by apoptosis or proteasome inhibition

(A) U87-MG cells were treated for 24h with DMSO, 2 μM Y102, or 2 mM Hydroxyurea. Cells were stained for Ɣ-H2AX, a double- and single-stranded DNA breaks marker (green). DAPI was used to counterstain cell nuclei (blue). Scale bar= 150µm. (B) U87-MG cells were treated for 48h with DMSO, 2 µM Y102, or 100 nM Doxorubicin. Cells were stained for cleaved-caspase 3, an apoptotic cell death marker (green). DAPI was used to counterstain cell nuclei (blue). Scale bar= 150µm (C) U87-MG cells were treated for 24h with DMSO or 2 µM Y102, 4 µM Y102, or for 2h with 10 µM Y102 or a combination of 1 µM Bortezomib and 10 µM MG-132 (iProt). Cell lysates were incubated with the dye MV-151, which binds to the active sites of proteasomal subunits. Samples were run on an SDS-PAGE gel. Fluorescence was measured by scanning the gel on a Typhoon scanner. Protein was transferred to nitrocellulose to probe for the loading control α-Tubulin. Densitometry analysis was used to determine the ratios of active proteosome subunits to α-Tubulin control. Experiment was repeated twice, and the image is representative of both experiments.

Journal of Cell Science • Supplementary information

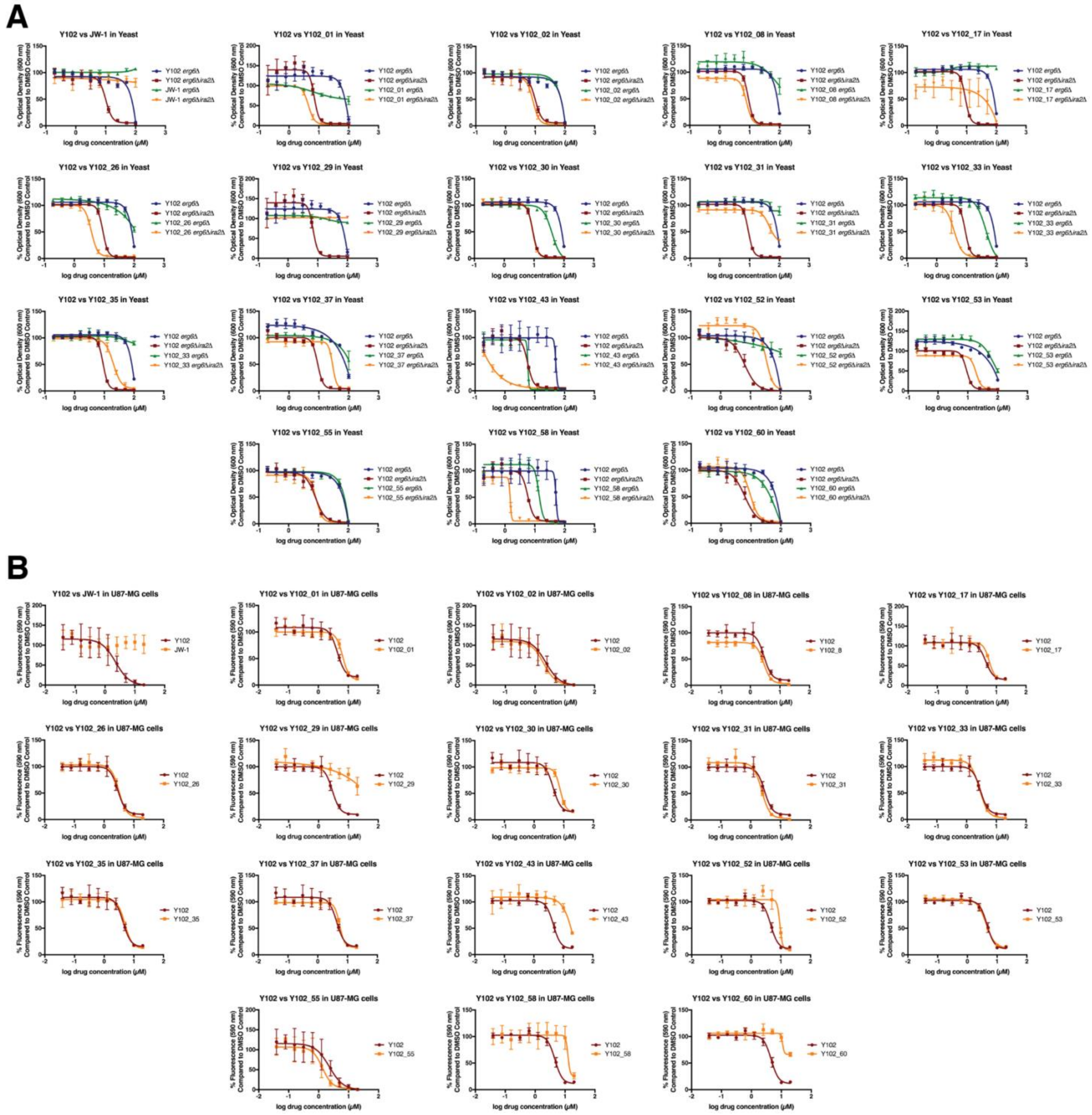

Fig. S3. Structure-activity relationship studies comparing the parent compound Y102 to twenty Y102 analogs

(A) Yeast were grown at 30°C starting at an OD_{600} of 0.05 with Y102 or various analogs of Y102 at increasing concentrations starting from 100μM. At 18h, OD_{600} was measured. (B) U87-MG cells were plated at 5,000 cells/well and treated for 72h with Y102 or various analogs of Y102 at concentrations ranging from 100 μM to 0.039 μM. Three hours prior to collection, cells were stained with alamarBlue. Plate fluorescence was read at an ex/em of 544 nm/590 nm.

Journal of Cell Science • Supplementary information

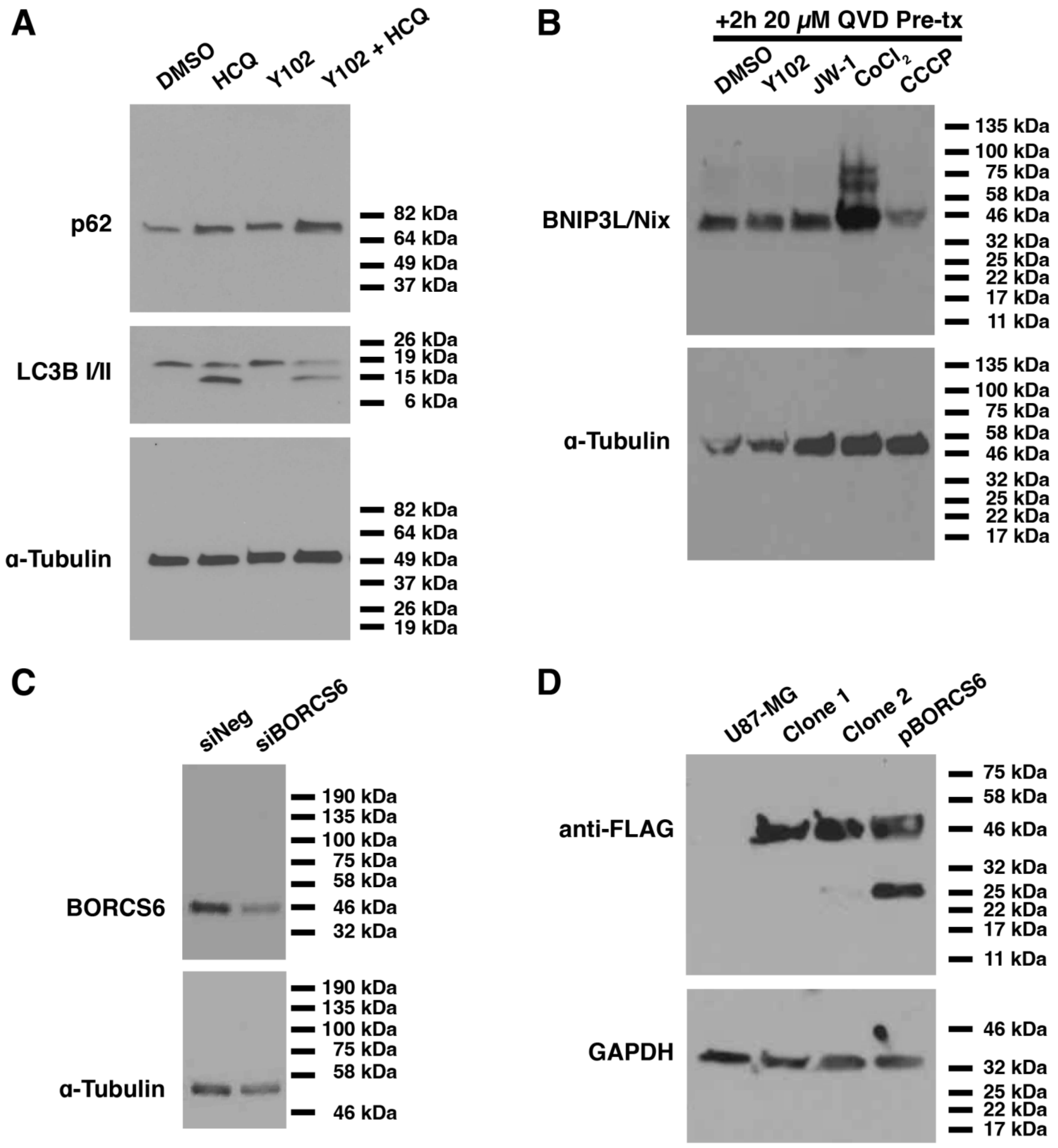

Fig. S4. Complete Western blot images for transparency

(A) Complete western blot of Figure 2C. U87-MG cells were treated for 12h with DMSO or 2 µM Y102 followed by an additional 12h +/- 50 µM HCQ prior to analysis of autophagy markers p62 and LC3B I/II. Tubulin serves as a loading control. (B) Complete western blot of Figure 3D. U87-MG cells were pre-treated with 20 µM QVD for 2 hours prior to 24-hour co-treatment with DMSO, 2 µM Y102, 2 µM JW-1 (not shown in Figure 3D), 100 µM $CoCl_2$, or 10 µM CCCP. Blot was probed for BNIP3L/Nix; tubulin served as a loading control. (C) Complete western blot of Figure 5A. U87-MG cells were treated with negative (siNeg) or BORCS6-specific (siBORCS6) siRNA for 72 hours. Blot was probed for BORCS6 and the loading control tubulin. (D) Complete western blot of Figure 7B. A western blot of lysates from U87-MG cells or multiple clones expressing FLAG-tagged BORCS6 was probed for BORCS6. Only the clone used (labeled pBORCS6) was included in Figure 7B; additional clones are labeled Clone 1 and Clone 2. GAPDH served as a loading control.

Journal of Cell Science • Supplementary information

Table S1. Analogs of tool compound Y102

JW-1 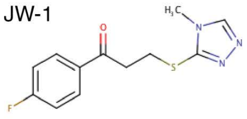	Y102_01 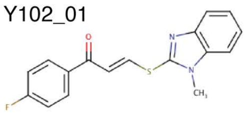	Y102_02 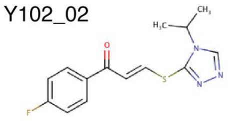
Y102_08 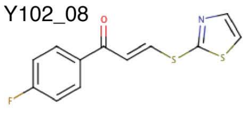	Y102_17 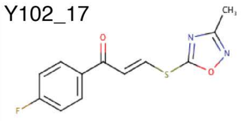	Y102_26 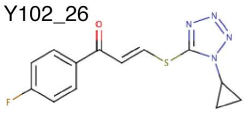
Y102_29 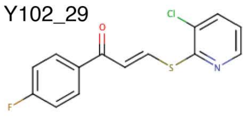	Y102_30 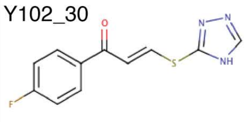	Y102_31 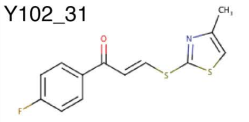
Y102_33 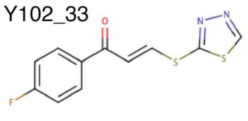	Y102_35 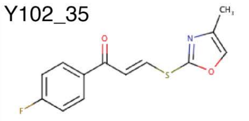	Y102_37 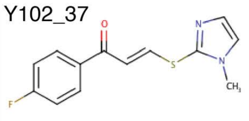
Y102_43 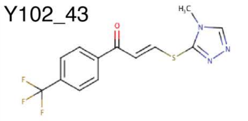	Y102_52 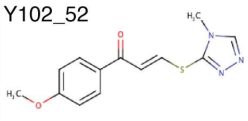	Y102_53 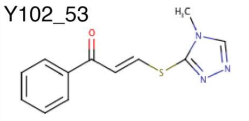
Y102_55 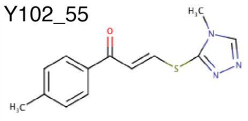	Y102_58 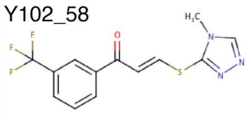	Y102_60 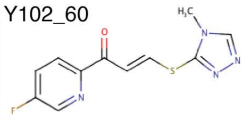

Table S2. Click-chemistry-aided mass spectrometry and CETSA results

Available for download at
https://journals.biologists.com/jcs/article-lookup/doi/10.1242/jcs.262343#supplementary-data

Journal of Cell Science • Supplementary information
